# Online Learning for Foot Contact Detection of Legged Robot Based on Data Stream Clustering

**DOI:** 10.3389/fbioe.2021.771415

**Published:** 2022-02-01

**Authors:** Qingyu Liu, Bing Yuan, Yang Wang

**Affiliations:** ^1^ Key Laboratory of Metallurgical Equipment and Control Technology, Ministry of Education, Wuhan University of Science and Technology, Wuhan, China; ^2^ Hubei Key Laboratory of Mechanical Transmission and Manufacturing Engineering, Wuhan University of Science and Technology, Wuhan, China; ^3^ Golden Leaf Production and Manufacturing Center of China Tobacco Henan Industrial Co., Ltd., Zhengzhou, China

**Keywords:** legged robot, contact detection, online learning, data stream clustering, Gaussian mixture model

## Abstract

Foot contact detection is critical for legged robot running control using state machine, in which the controller uses different control modules in the leg flight phase and landing phase. This paper presents an online learning framework to improve the rapidity of foot contact detection in legged robot running. In this framework, the Gaussian mixture model with three sub-components is adopted to learn the contact data vectors corresponding to running on flat ground, running upstairs, and running downstairs. An online data stream learning algorithm is used to update the model. To deal with the difficulty in obtaining contact data at landing moment online, a “trace back” module is designed to trace back the contact data in the memory stack until the data meet with the probability contact criterion. To test if the foot is in contact with the ground, a projection method is proposed. The acquiring data vector during the leg flight phase is projected onto an independent random vector space, and the contact event is triggered if all projected random variables fall within 1.5*σ* of the corresponding Gaussian distribution. Experiments on a legged robot show that the presented algorithm can predict the foot contact 16 ms in advance compared with the prediction using only leg force, which will ease the controller design and enhance the stability of legged robot control.

## Introduction

The ability to negotiate unstructured terrain is the most significant advantages of the legged robot compared with wheeled and tracked vehicles. Due to the discrete foot point characteristic in legged locomotion, like the human and other legged animals, the robot goes through a series of foot contact in locomotion. Based on different foot contact states, a finite state machine is usually adopted to identify the gait phases, and then different control modules will plan the leg motion trajectory to balance the robot. Thus, robust perception of the foot contact arises as a crucial ability in legged robot control. Though a force sensor mounted on the foot could be a straightforward solution (Wagner et al., 2017), it is easily damaged due to the foot–ground impact and the unknown rough terrain. Furthermore, the foot force sensor would increase the inertia of the leg.

Rather than using an indirect perception method like visual sense (Jiang et at., 2021), the endpoint force estimator is a classical approach to detect the endpoint contact state (Morinaga and Kosuge, 2003; Ortenzi et al., 2016; Camurri et al., 2017). To avoid calculating the acceleration of the joint angle, a more feasible approach based on the generalized momentum is adopted (De Luca et al., 2006; Manuelli and Tedrake, 2016). Considering the floating nature of the mobile robot trunk, Flacco et al. (2016) developed a formulation of the residual based on the floating-base dynamics of the humanoid to estimate the external force. A further extension to the multi-contact situation was done by Manuelli and Tedrake (2016) on an atlas robot. Some other works can be used as reference (Haddadin et al., 2008; Li et al., 2019; Dong et al., 2020; Wang et al*.*, 2020; Yousefizadeh and Bak, 2020). The main drawback of these contact detection methods is that they only use the robot dynamic information, such as joint angle and angular velocity. These data are always noisy, and the kinetic parameters of the robot may change as the robot runs for a long time, which would degrade the detection performance.

To make a more robust contact prediction, data fusion in the probability framework was introduced by Hwangbo et al. (2016). This approach fused dynamics, differential kinematics, and kinematics using a hidden Markov model (HMM) to infer the contact state. Kim and Lee (2017) used the IMU data of the human body, leg, and foot to predict the foot contact. The Kalman filter is another framework to fuse the acquired information (Miezal et al., 2017; Yang et al., 2019). Camurri et al. (2017) used approximate ground reaction forces as input to a contact probability prior to determining if the foot is fully in contact with the ground. A most impressive work in this direction was presented by Bledt et al. (2018b), and the contact detection algorithm had been applied to the MIT Cheetah 3 robot (Bledt et al., 2018a). They used the extended Kalman filter to fuse the estimated leg force, gait phase, and leg height and achieved very high detection accuracy.

Though the current data fusion methods work well in foot contact detection, some model parameters need to be selected very carefully, and the robustness to robot kinetic parameters change is unknown. The learning approach provides a promising solution to this challenge. Rotella et al. (2018) employed fuzzy C-means (FCM) clustering to differentiate contact from leaving states using the contact wrench and IMU data. Piperakis et al. (2019) directly learned the gait phase by clustering, in which foot contact detection is an implied process. But both of the approaches need the measuring forces, and the clustering process is completely off-line. Ma et al. (2019) and Lin et al. (2021) trained a Gaussian mixture model (GMM) to cluster the contact data set. However, they both assumed the availability of a very sophisticated force and visual perception system. Neural networks are also used to learn robot contact (Sharkawy et al., 2020). However, all of these learning algorithms were done off-line. In real legged robot application, the robot should deal with impact and unknown rough terrains, so a contact detection method which can adapt to changing robot parameters and environments is desirable.

The main contribution of this paper is to present an online learning framework for foot contact detection of a legged robot. The detection algorithm adapts the model parameters to different terrains for a fast and accurate detection. For online learning, a “trace back” scheme for an online contact data acquisition method is proposed. A projection technique is adopted for a fast prediction of the contact states based on the GMM.

## Materials and Methods

### Robot

**FIGURE 1:**
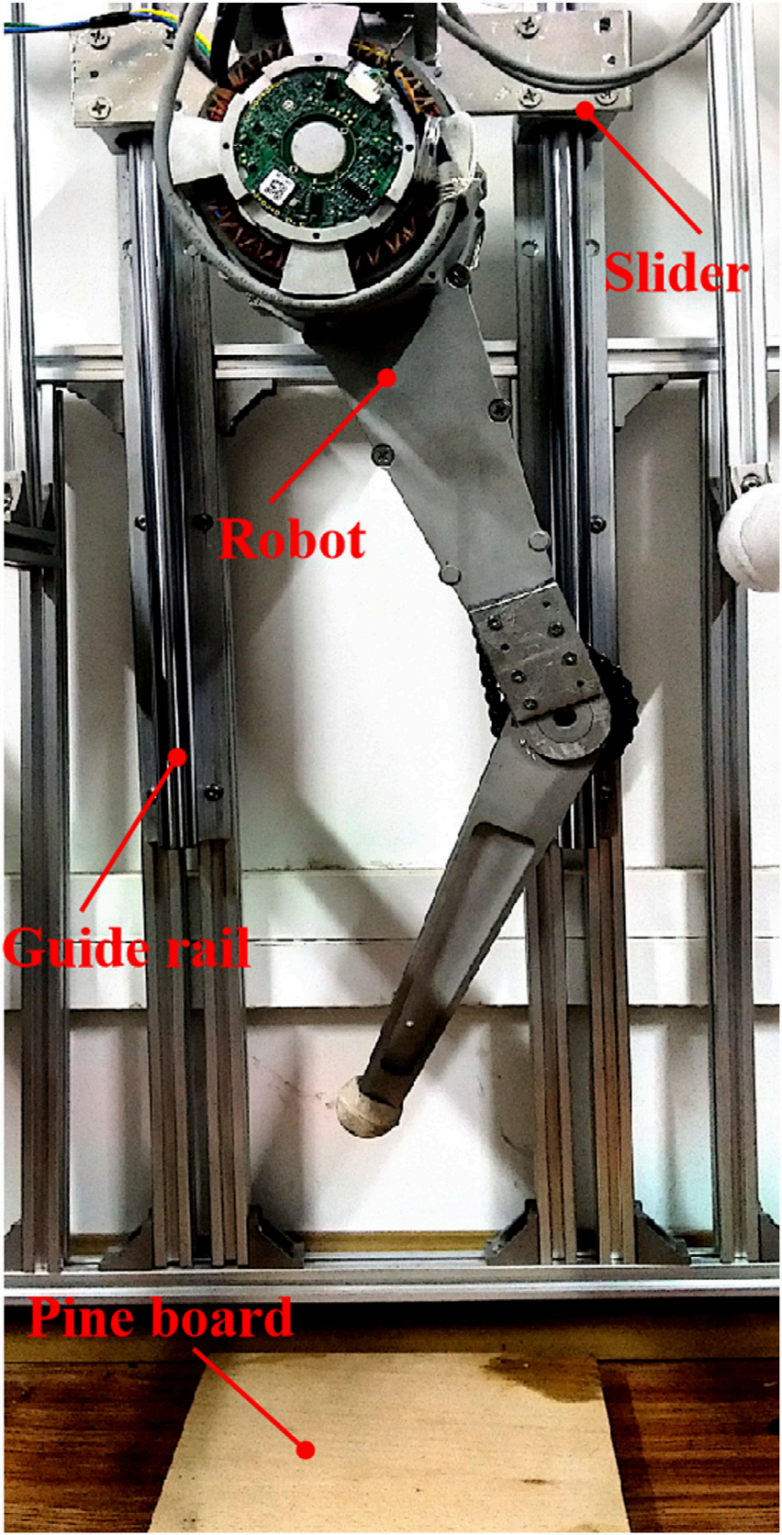
Experimental platform.

The proposed foot contact prediction framework is verified on an electric motor actuated hopping robot DynJump, as shown in Figure 1. The hip joint is actuated directly by the motor while the knee joint is actuated through train driving. The parameters of the robot are listed in Table 1.

**TABLE 1:** Parameters of the robot.

Item	Type/Value
Total mass	5 kg
Structural material	Aluminum alloy
Upper leg length	0.25 m
Lower leg length	0.24 m
Foot material	Rubber
Knee train gear ratio	2.1
Power supply	48 V
Motor mass	1.5 kg
Max motor stall torque	48.8 Nm at 48 V
Continuous motor stall torque	20.5 Nm at 8.4 V

To focus on the study of foot contact prediction, the robot hopping motion is constrained in a vertical direction through a guide rail. The control board communicates to the motor driver through the CAN protocol at 250 Hz.

### Robot Control

**FIGURE 2:**
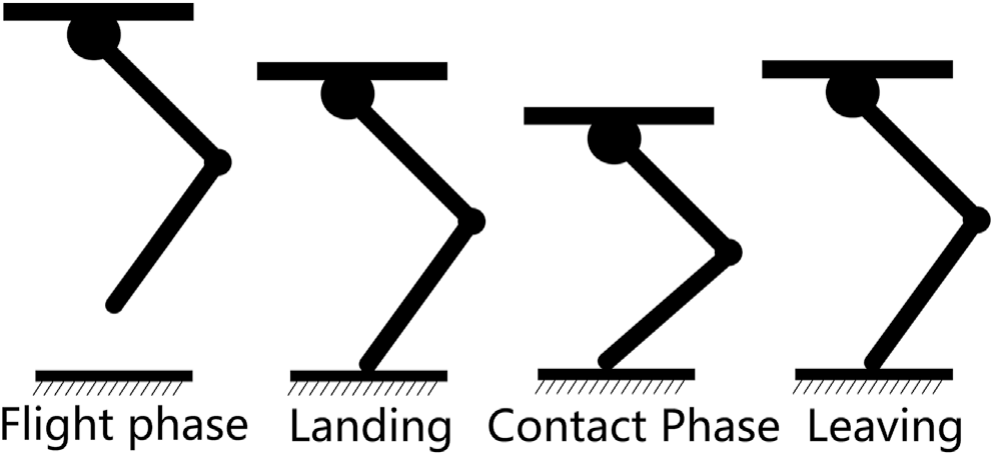
Robot states in hopping.

As we only focus on verifying the foot contact detection, a classical finite state machine is adopted as a high-level control scheme. The robot states are divided into the flight phase and contact phase based on the foot contact states as shown in Figure 2.

In the flight phase, a pre-calculated foot point trajectory is tracked through a position control scheme. The controller would bring the leg length to a predefined nominal leg length and leg angle for the next landing. After the leg landing, the leg motion is planned based on a virtual spring–mass model (Piovan and Byl, 2015). The detection algorithm for the state switching control will be discussed in the following section. The robot trunk vertical velocity is estimated at the landing moment to initialize the calculation of the robot trajectory after landing. As the robot is only subjected to the force of gravity in the flight phase, the initial velocity can be derived as

$$v_{\text{c}}=v_{0}-g\cdot \left(t_{\text{c}}-t_{0}\right)$$ (1)

where *v*_c_ and *t*_c_ are the velocity and time at the moment the foot contacts the ground and *g* is the gravity constant. For determining the exact moment when the foot leaves the ground, we actively shorten the leg at the later stage of the contact phase, and the corresponding time and robot vertical velocity are recorded as *t*_0_ and *v*_0_.

### Probability Contact Prediction Model

As the contact is an impact between the robot and the environment, variables associated with the interaction can be used to indicate the contact state of the foot. Though foot force can reflect the physical interaction between the foot and the ground, we can only estimate it based on the dynamics of the leg due to the absence of the foot force sensor. In robot dynamics, sensor noise and transmission clearance may introduce errors in foot force estimation. Furthermore, the kinetic parameters are always changing and unknown, so it is not very reliable if we only used the foot force to predict the contact. Although we can adopt a higher threshold to increase the reliability, a larger time delay in contact detection will degenerate the robot control performance.

The foot height and the gait cycle represent the kinematic interaction between the robot and the environment. For a running gait of the robot, like trotting gait, the robot will completely leave the ground during running, and the accurate foot height is difficult to obtain based on proprioceptive perception. Similarly, the gait cycle is highly dependent on the terrain and gait. So limited prediction performance would be obtained if we used kinematics information only.

Based on the analysis above, an indicator vector ***s*** = [f_y_h_foot_t_g_]^T^ is defined to estimate the contact probability of the foot contact. Here f_y_ is the vertical foot force, h_foot_ is the foot height relative to the leaving ground level, and t_g_ is the gait cycle. Though Kalman filter is suitable for fusing the measuring data, a method which can learn from real data online would have advantages in environment adaption and be more robust to robot kinetic parameter uncertainty.

#### The GMM

To deal with different types of terrain, a GMM is adopted. Based on the insight into the legged locomotion in different terrains, we divide the terrains into three categories, and each is modeled using a basic Gaussian model, which is a sub-component of the GMM.

**FIGURE 3:**
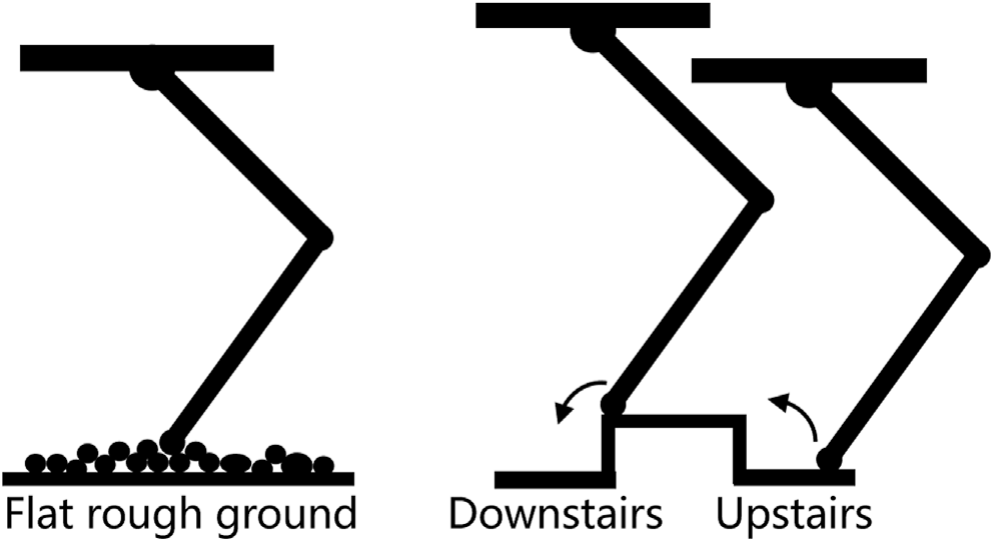
Three categories of terrain in hopping.

One basic Gaussian model accounts for the flat ground that has different roughness, and the others correspond to the robot going upstairs and downstairs as shown in Figure 3. We can easily obtain the foot contact probability of incoming data as

$$p\left(x|\Theta \right)=p\left(x|\pi ,\mu ,\Sigma \right)=\overset{K}{\underset{k=1}{\sum }}\pi_{k}N\left(x|\mu_{k},\Sigma_{k}\right)$$ (2)

where the Θ is the model parameters, *π* is the weight of the particular Gaussian model, ***μ*** is the mean value, ***Σ*** is the covariance matrix, *K* is the number of the sub-component of the GMM. And the posterior probability of the data *x* belonging to component *k*, which is also known as responsibilities *p*(*k*| ***x***), is given by Bayes’ theorem as

$$\gamma_{k}\left(x\right)=p\left(k|x\right)=\frac{\pi_{k}N\left(x|\mu_{k},\Sigma_{k}\right)}{\sum_{l}\pi_{l}N\left(x|\mu_{l},\Sigma_{l}\right)}$$ (3)

#### Indicator Vector Calculation

*Foot point force*: The foot force is a primary indicator of the contact. A generalized momentum method as in De Luca et al. (2006) is used to estimate the joint torque $\;{\hat{\tau }}_{e}\;$ corresponding to the external force on the foot point; it follows that

$${\hat{\tau }}_{e}=\lambda p\left(t\right)-\lambda \overset{t}{\underset{0}{\int }}\left(\tau +C^{T}\dot{q}-g+{\hat{\tau }}_{e}\right)dt$$ (4)

where $q\in R^{n}$ is the joint angles, *n* is the number of degrees of freedom, ***p*** = ***Mq*** is the generalized momentum, and $C\in R^{n\times n}\;$ is a factorization of the Coriolis terms, which makes $\dot{M}-2C\;$ a skew-symmetric matrix. Then the estimated foot point force ${\hat{f}}_{e}={\left[f_{x}\;f_{y}\right]}^{T}\;$ can be calculated using the Jacobian matrix *J* as

$${\hat{f}}_{e}=J^{T}{\hat{\tau }}_{e}$$ (5)

We hypothesize that there is no singular position during the flight phase, as the leg is always in a bent position during hopping.

*Foot height*: As the terrains are unknown, we estimate the foot height relative to the leaving ground level. If the ground is flat, the foot is expected to land when the height is back to zero, and if there is a step, the height at landing would be significantly higher or lower than zero. The foot height can be expressed as

$$h_{foot}=v_{0}t-\frac{1}{2}gt^{2}-\left(l_{t}-l_{0}\right)$$ (6)

where *v*_0_ is the initial vertical speed of the robot trunk, *g* is the gravity acceleration, *t* is the time relative to the leaving ground moment, *l*_*t*_ is the leg length function depending on the joint angle, and *l*_0_ is the initial leg length. We should note that as we assume that the robot runs in a sagittal plane and the pitch angle is constrained, *v*_0_ can be derived by taking the derivative of the function *l*_*t*_.

*Gait cycle*: In a stable gait, the robot movement is always periodic, and therefore, the foot contact event is triggered periodically. In the viewpoint of probability, a cyclic movement means that a contact event most likely happens after a specific period of time from the previous foot contact. Thus, a gait time is adopted as one of the indicators for foot contact prediction. The gait cycle is calculated as in (7).

$$t_{g}=t-t_{\text{c}}$$ (7)

where *t* is the robot running time and *t*_c_ is the time of the previous foot contact.

#### Foot Contact Prediction

When the robot is in the flight phase, the robot should estimate the contact state based on the sampling indicator vector calculated in previous section. With the GMM, the probability density of the newly coming data vector can be obtained. However, it is tedious to integrate the GMM density function to gain the distribution function, which means that it is computationally inefficient to infer the contact state by the probability density of the random vector. In this paper, we project the indicator vector along the direction which decouples the random vector into three independent random variables.

The projection matrix is determined using the covariance matrix in GMM (Horn and Johnson, 2012). As the covariance matrix is a semi-definite symmetric matrix, there exists a matrix ***C*** that satisfied ***D*** = ***CΣC*** ^T^, where ***D*** is a diagonal matrix with diagonal entries $\;\sigma_{1}^{2}$ , $\;\sigma_{2}^{2}$ , and $\;\sigma_{3}^{2}$ and ***Σ*** is the covariance matrix. The matrix C can be determined using the elementary transformation. Then the random vector can be projected as

$$s_{p}=\mathrm{Cs}$$ (8)

And the mean of the random variable vector of ***s***_p_ is ***Cμ***, and *μ* is the mean before projection. The three elements in vector ***s***_p_ are three independent random variables, and the variances are $\;\sigma_{1}^{2}$ , $\;\sigma_{2}^{2}$ , and $\;\sigma_{3\;}^{2}$ . So we define the contact vector set S_c_ as

$$S_{\text{c}}=\left\{s\;|\;s_{p}=\mathrm{Cs},\;|s_{p1}-\mu_{1}|<1.5\sigma_{1}\;,\;|s_{p2}-\mu_{2}|<1.5\sigma_{2}\;,\;|s_{p3}-\mu_{3}|<1.5\sigma_{3}\right\}$$ (9)

where $s_{p}={\left[s_{p1},\;s_{p2},\;s_{p3}\right]}^{T}$ and $\mu ={\left[\mu_{1},\;\mu_{2},\;\mu_{3}\right]}^{T}$ . The definition means that if the three projected random variables are all located in 1.5*σ* of the Gaussian probability density function, we assume that the leg contact event is triggered.

### Initial Model Parameter Training

Though we expect that the robot can adapt to different terrains online, an off-line learning to obtain a group of initial parameters can speed up the online learning process. We used the classic two-step expectation maximization (EM) method for training. The two steps can be summarized as follows.

*E-step*: The responsibilities can be computed as

$$\gamma_{ik}=\frac{\pi_{k}p\left(x_{i}|\theta_{k}^{t-1}\right)}{\sum_{k^{\prime }}\pi_{k^{\prime }}p\left(x_{i}|\theta_{k^{\prime }}^{t-1}\right)}$$ (10)

*M-step*: The parameters in the Gaussian component can be calculated as

$$\mu_{k}=\frac{\sum_{i}r_{ik}x_{i}}{r_{k}}$$ (11)

$$\Sigma_{k}=\frac{\sum_{i}r_{ik}\left(x_{i}-\mu_{k}\right){\left(x_{i}-\mu_{k}\right)}^{T}}{r_{k}}$$ (12)

And for the next iteration,

$$\pi_{k}=\frac{1}{N}\underset{i}{\sum }r_{ik}=\frac{r_{k}}{N}$$ (13)

**FIGURE 4:**
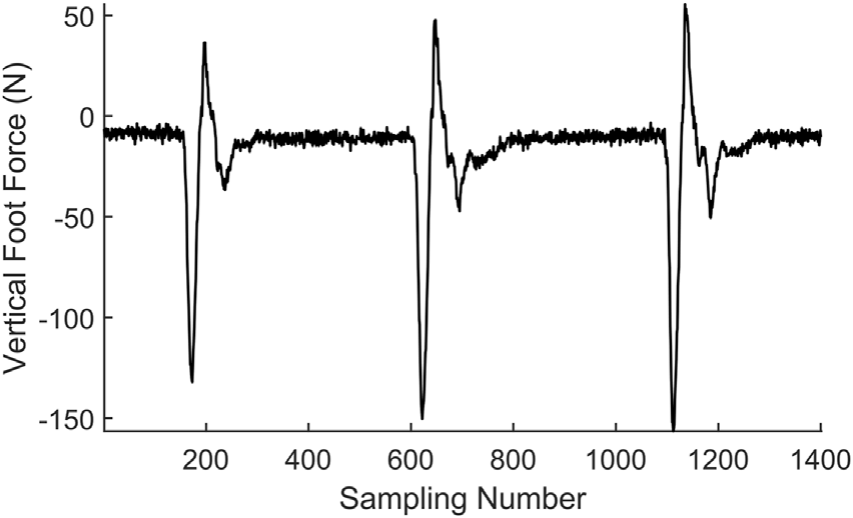
Vertical foot force in free-fall experiment.

In the data training procedure, a series of hopping experiments were conducted for data collection. The robot hopped on flat ground, upstairs, and downstairs. A high landing force threshold was adopted for reliable contact detection as the GMM had not yet been established. The threshold was determined based on the estimated foot force in a robot free-fall experiment, as shown in Figure 4. The corresponding fly phase time, estimated leg force, and foot height were simultaneously recorded at 200 Hz by the control board. We found that there was a non-negligible time delay in landing detection due to the high landing force threshold. We manually selected the contact data on the force cure after we collected all the experimental data. For each terrain, 30 contact data points were collected, and the learning process was done in the MATLAB 2018b environment.

### Online Model Adaption for Different Terrains

As the terrain is unknown and the robot parameters may change over time, an online GMM adaption is desirable for robot control. As there are many online data stream clustering methods (Kokate et al., 2018), we would focus on dealing with the outlier point caused by the chatter effect, which balances the response time and model stability. Another problem in online clustering for leg contact detection is how to identify the sensory data corresponding to the foot–ground contact moment without the foot force sensor or external force sensors mounted on the ground. We cannot use the predicted contact results of GMM as the data are used for training the prediction model. A likelihood criterion of the foot contact event is used to find the exact contact data. This method would lead to a large time delay that cannot be used for contact prediction, but the data can then be used for online training of the GMM.

Here we use a modified version of SWEM (Dang et al., 2009). Three independent data sets are established corresponding to three GMM components. When the contact data were acquired, the probability density of the data in each of the three GMM components is calculated. The contact data would be put into the data set corresponding to the GMM component, which has the maximum probability density. When the number of newly arriving data in any of the three data sets reaches 5, the GMM will be updated as in the algorithm presented in this section.

We define that *g*(*10*) is the GMM of the data from the beginning 1 to *N*, and the newly arriving data are x_N+1_, ⋅⋅⋅, x_N+M_ as in Song and Wang (2005). Different from many other applications, only one contact datum is available in one gait cycle, so we define a time window with five data points, and only one basic Gaussian model is used to model the five data points. The maximum likelihood method is used to estimate the parameters.

$$\frac{\partial \text{ln}L\left(\theta \right)}{\partial \theta_{j}}=0$$ (14)

where *j* is the number of the parameters and L(*θ*) is the likelihood function, $L\left(\theta \right)=\overset{n}{\underset{i=1}{\prod }}f\left(x_{i},\theta \right)$ . This calculation is conducted for every five newly arriving data. When we obtain the mean and variance matrix of the Gaussian model, a T^2^ statistic testing for the equality of the mean to the existing mean value in GMM is conducted (Song and Wang, 2005). Once the mean value is tested to be equal to any existing basic model, the Gaussian model will be merged to the corresponding basic Gaussian sub-model in GMM with the rule

$$\mu =\frac{N\pi_{j}\mu_{j}+M_{k}\mu_{k}}{N\pi_{j}+M_{k}}$$ (15)

$$\Sigma =\frac{N\pi_{j}\Sigma_{j}+M_{k}\Sigma_{k}}{N\pi_{j}+M_{k}}+\frac{N\pi_{j}\mu_{j}\mu_{j}^{T}+M_{k}\mu_{k}\mu_{k}^{T}}{N\pi_{j}+M_{k}}-\mu \mu^{T}$$ (16)

$$\pi =\frac{N\pi_{j}+M_{k}}{N+M_{k}}$$ (17)

where ***μ***_j_ and ***Σ***_j_ are the mean and covariance of the *j*th sub-component of GMM; *π*_j_ is the weight of the *j*th sub-component in GMM; ***μ***, ***Σ***, and *π* are the new mean, covariance, and weight, respectively; *N* is the total number of data that have been used for update; and *M*_*k*_ is the data number in the time window.

If the mean value is tested to be different from any basic Gaussian model in the GMM, the new Gaussian model will be labeled as a temporary outlier Gaussian model. For the outlier Gaussian model, it will also be merged to the nearest basic Gaussian sub-model in GMM, but a fading rule will be applied to it until it is determined as an outlier model or a shift of the existing basic Gaussian model. The merging procedure will be carried out in two steps.

Firstly, the temporary outlier Gaussian model will be merged to the existing outlier Gaussian model with the fading rule as

$$\mu =\frac{\lambda M_{0}\mu_{0}+M_{k}\mu_{k}}{M_{0}+M_{k}}$$ (18)

$$\Sigma =\frac{\lambda M_{0}\Sigma_{0}+M_{k}\Sigma_{k}}{M_{0}+M_{k}}+\frac{\lambda M_{0}\mu_{0}\mu_{0}^{T}+M_{k}\mu_{k}\mu_{k}^{T}}{M_{0}+M_{k}}-\mu \mu^{T}$$ (19)

where ***μ***_0_ and ***Σ***_0_ are the mean and covariance of the existing outlier Gaussian model, respectively; ***μ***_k_ and ***Σ***_k_ are the mean and covariance of the temporary outlier Gaussian model, respectively; ***μ*** and ***Σ*** are the new mean and covariance, respectively; *M*_0_ is the total number of data in the outlier set; and *λ* is the fading factor.

We should note that this procedure will be carried out in every model update no matter if the data are outliers or not. When the mean of the newly coming data is tested equal to the existing mean in GMM, the ***μ***_k_ and ***Σ***_k_ in (18) and (19) are set to zero; thus, the existing ***μ***_0_ and ***Σ***_0_ are faded down by factor *λ*.

Secondly, the resulting new outlier model will be merged with the nearest basic Gaussian component. But the parameters ***μ***, ***Σ*,** and *π* of the present basic Gaussian component are reserved in the memory. When the next time window data come, if it is tested equal to any basic Gaussian component, the model will be merged as formulas (15)–(17), or if it is tested as an outlier model, the model will be moved to the outlier set and merged to the existing outlier model as formulas (18) and (19). Then the merged model will be merged with the nearest basic model.

When the outlier data situation emerges three times in succession, we assume that a concept shift happens, which means that the terrain or gait changes. Then the corresponding outlier data will be moved out of the outlier set and merged to the nearest basic component. The resulting GMM is considered as a new initial model for the newly arriving data. On the other hand, if the outlier data situation does not emerge in three consecutive time windows, the mean and the covariance will soon decrease to zeros due to the fading factor.

### Online Contact Data Acquisition

As we learn the contact model online without the force sensor mounted on the ground or foot, identifying which data are the contact data becomes a difficult task. Though the contact prediction module presented above will give an indication, we need to get the data in another way in order to update the prediction model and improve the prediction performance.

To collect the contact data, a “trace back” module is proposed. The algorithm includes two steps: firstly, we would detect the contact event using the GMM or leg force threshold, and then we would trace back from the contact event time to find the exact contact data.

**FIGURE 5:**
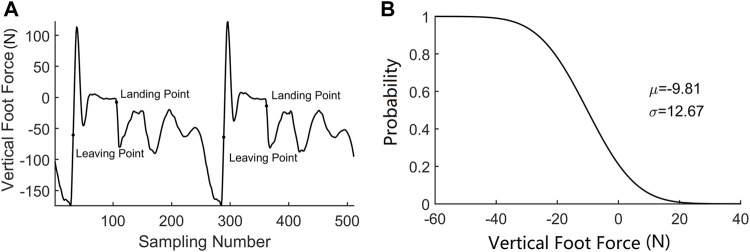
**(A)** Foot force during hopping. **(B)** Contact force distribution function.

Two situations are considered in the contact event detection: one is when the contact is triggered by the GMM, and the other is when the contact is triggered by the estimated foot force. It is obvious when the contact event happens in the first situation as the GMM will give an indication. However, when the robot encounters a new terrain or if the robot is unstable, the estimated foot force will firstly trigger the contact event. A typical estimated foot force trajectory during hopping is shown in Figure 5A. A fact we should note is that the estimated foot force changes to positive when the foot just leaves the ground, which is inconsistent with the physical truth as the foot force should either be negative or zero under the definition of foot force direction in this paper. This estimation bias occurs when the leg shortens rapidly to force the robot to enter the flight phase, which will converge to zero in about 20 sampling cycles before the next foot landing. A time criterion is added to filter this abnormal condition. A likelihood technique is used to determine if the contact event is triggered. The distribution function of the contact force is used to verify the contact probability at each time stamp. We test five consecutive foot force data to ensure that the contact event detection is reliable. The adopted distribution function is shown in Figure 5B, and the contact event criterion is defined in (20).

$$1-F\left(x_{p}\right)\cdot F\left(x_{p-1}\right)\cdot F\left(x_{p-2}\right)\cdot F\left(x_{p-3}\right)\cdot F\left(x_{p-4}\right)<0.01$$ (20)

where x_p_ is the coming data corresponding to the present sampling time and x_p−n_ is the data at n previous sampling time. The criterion means that if the misjudgment probability is below the threshold in a five-sample time window, we assume that the foot is in contact with the ground in the present sampling time. During robot running, a sliding window containing five data points is built, and the data in the sliding window will be tested if they satisfy the criterion in (20) in every control cycle. As the contact test is a conservative estimation, the resulting contact moment is several time steps after the leg contact, which is the reason why we cannot use this method to indicate the leg contact event for robot control.

**FIGURE 6:**
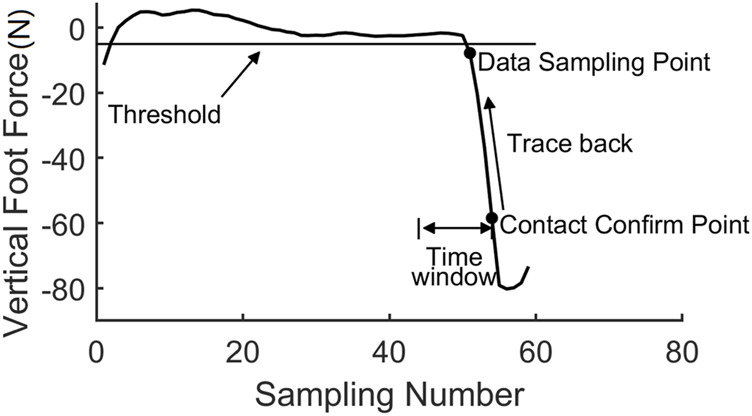
Trace-back scheme.

As shown in Figure 6, as the lower point on the curve satisfies the contact criterion, a contact event happens. Once a contact event is confirmed, a “trace back” method is invoked to find the exact contact time moment. The contact probability is calculated in reverse order along the curve from the present data until the contact probability of the data is less than 0.5. The last data point in this procedure, with a probability of more than 0.5, will be adopted as the contact data. The “trace back” algorithm is shown in Table 2. In the algorithm, if the contact event is triggered, i.e., the trigger flag variable *trigger* = 1, a “trace back” procedure is employed to calculate the contact probability of *F*_p−k_ until the corresponding probability is less than 0.5.

**Algorithm 1:** Table 2 Trace-back Algorithm

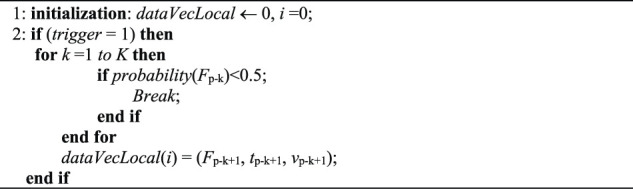

Once five contact data are collected, a local Gaussian model is built using (14). To determine which sub-component of the GMM is to be updated, a testing procedure is adopted to determine if the mean value of the new arriving data is equal to any of the existing sub-models. Here, we use Hotelling’s T^2^ test, which is applicable for multivariate normal data. The H0 hypothesis is the mean value of the contact data *µ* = *µ*_j_, where *µ*_j_ the is one of the mean values of the sub-component of the GMM. When the sampling size is small, the T^2^ statistics can be expressed as

$$T^{2}∼n{\left(\bar{x}-\mu_{j}\right)}^{T}\Sigma_{0}^{-1}\left(\bar{x}-\mu_{j}\right)$$ (21)

where *n* is the number of the samples, ***Σ***_0_ is the covariance matrix of the sub-model in GMM, and $\;\bar{x}$ is the mean of the samples. If the H0 hypothesis is satisfied, we can derive that

$$\frac{n-d}{d\left(n-1\right)}T^{2}∼F_{d,n-d}$$ (22)

where F_d,n−d_ refers to an *F* distribution with *d* numerator degrees of freedom and *n* − *d* denominator degrees of freedom, *n* is number of the samples, and *d* is the dimension of the data vector. We can refer to the *F* distribution table to find out if the derived random variable in (22) obeys the *F* distribution, thus determining whether the H0 hypothesis is true. If the H0 hypothesis is true, the data will be used to update the GMM; otherwise, the data would be considered as outlier points if the mean is not equal to any means of the sub-components of the GMM.

## Results

### Hopping With Foot Force Contact Detection

**FIGURE 7:**
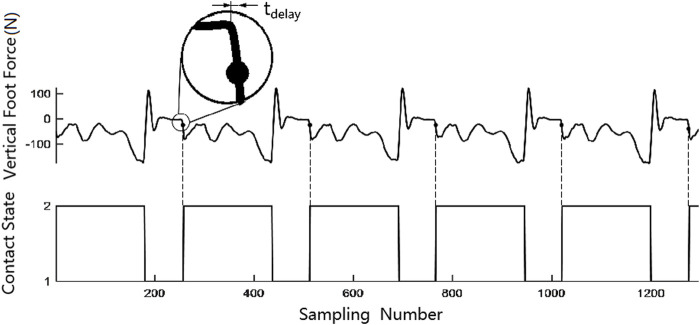
Contact detection with foot force on flat ground.

**FIGURE 8:**
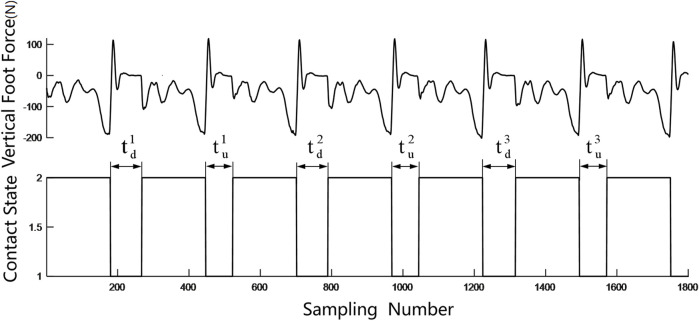
Contact detection with foot force in hopping upstairs and downstairs.

For comparison, the robot hopping data of contact detection with only foot force were collected. The foot force threshold was 40 N in the hopping experiment, which means that the landing control program would be triggered when the estimated foot force exceeded 40 N. The threshold was selected based on experimental data, which could prevent an incorrect estimation of the contact state due to the evaluated error of the foot force and the noise in data measurement. A time criterion was added to the contact detection in this experiment, in which the contact event was triggered only 50 ms after the leg left the ground. Hopping data on flat ground are shown in Figure 7. Data of hopping upstairs and downstairs are shown in Figure 8; the *t*_delay_ is the time delay between the leg in contact with the ground and the leg force exceeding 40 N; *t*_u_ and *t*_d_ are the flight times of hopping upstairs and downstairs, respectively. The time delay is unavoidable because we have to ensure that the contact is not triggered by the noise or evaluated error. In hopping experiments, the time delay is about 16 ms.

### Initial GMM Training Through Hopping Data

To establish the initial GMM, we collected three groups of contact data, corresponding to hopping on flat ground, hopping upstairs, and hopping downstairs. In hopping-upstairs and hopping-downstairs experiments, we placed a wood plank under the foot when the robot was in the flight phase to mimic hopping upstairs and took away the plank after the robot took off to mimic hopping downstairs. The thickness of the wood plank was 1.5 cm, which was limited to the ability of the motor and transmission system. Though the height is relatively small compared with the size of the leg, it does verify the effectiveness of the prediction method as the higher the stair is, the farther different contact data in the phase space are away from each other, which will ease contact detection.

**FIGURE 9:**
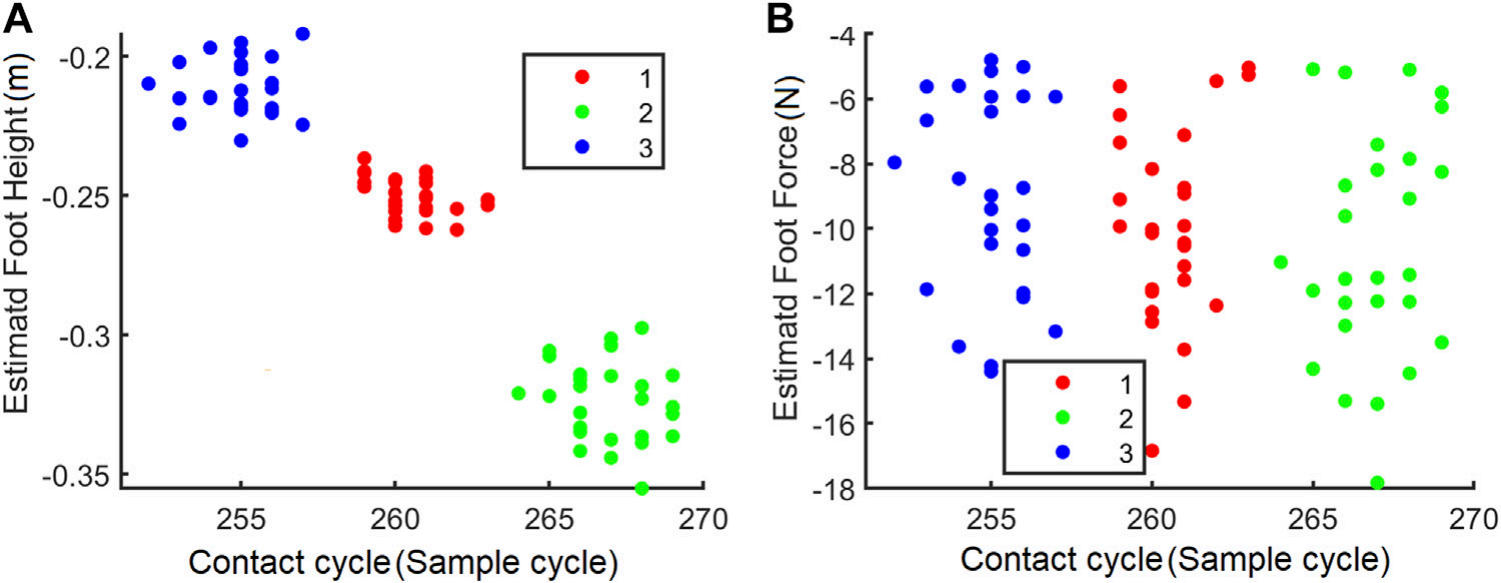
**(A)** Initial Gaussian model visualized by contact cycle and foot height. **(B)** Initial Gaussian model visualized by contact cycle and foot force.

The contact data were processed in MATLAB 2018b and were selected manually in this stage. Then, the contact data were clustered using the *fitgmdist* function in MATLAB with a component parameter of 3. The classification result is shown in Figure 9.

### Contact Prediction Using GMM

**FIGURE 10:**
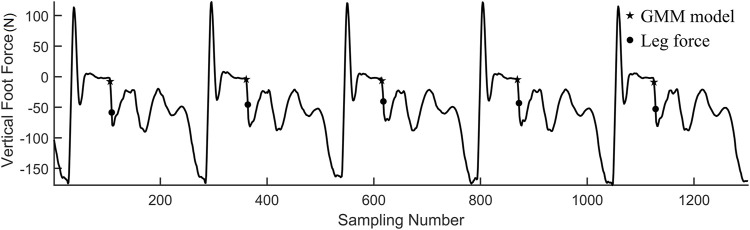
Hopping on flat ground with GMM contact prediction.

After the learning process, the GMM could predict the contact time more accurately, as shown in Figure 10. In each control cycle, the maximum probability of coming data in the three components is calculated first. Then, the mean value of the coming data vector will be projected into a space where the three projected variables are independent as in (8). If all of the values are within 1.5*σ*, we believe that the contact happens. By using the actual contact data to renew the GMM, the model detects the contact event as soon as the leg lands.

**FIGURE 11:**
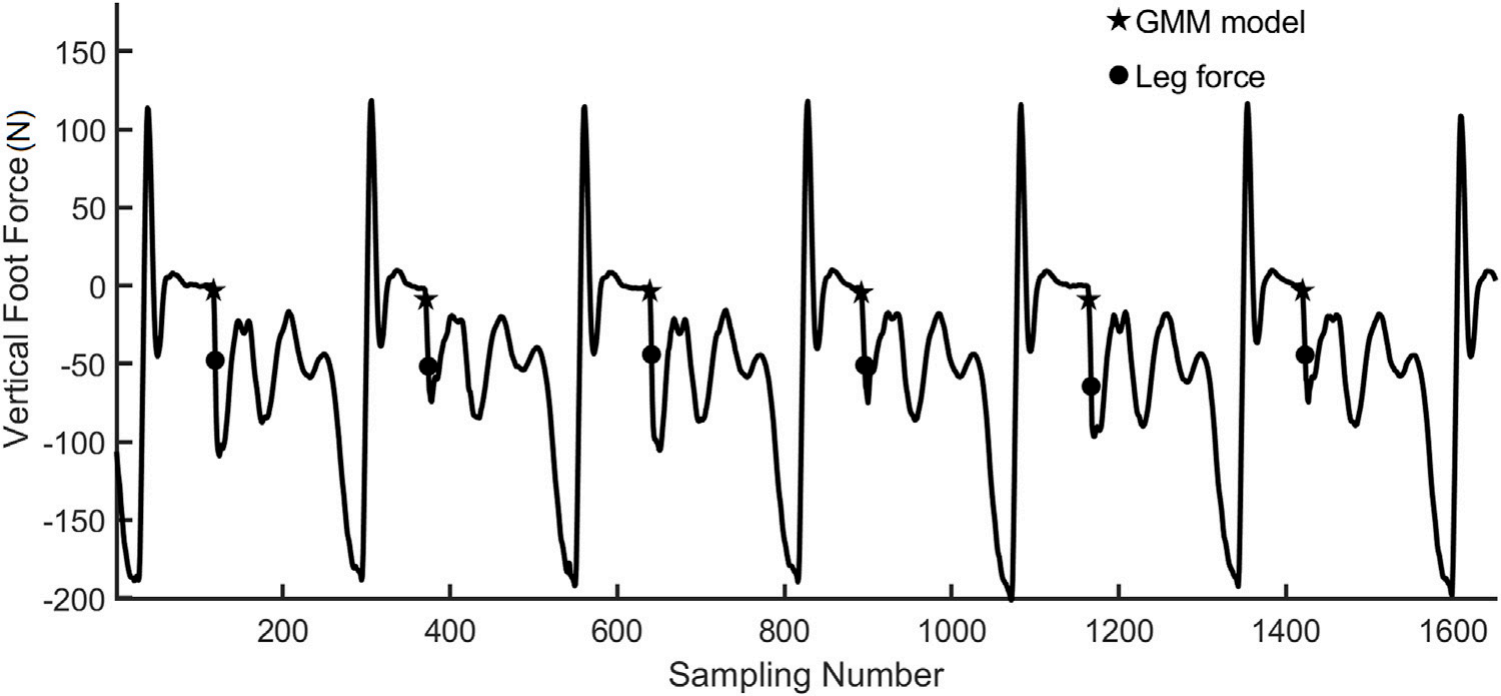
Hopping upstairs and downstairs with GMM contact prediction.

The GMM predicted the contact in stepping upstairs and downstairs, which was also more precise than the prediction using contact force, as shown in Figure 11.

**FIGURE 12:**
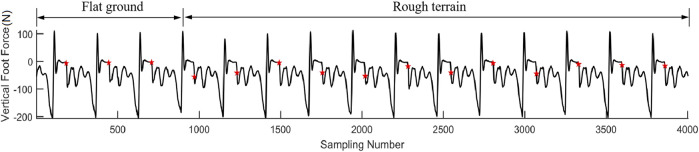
Hopping on roughness-changing ground.

The main advantage of the learning method is that it can adapt to the change in terrains. In a terrain-changing experiment, the robot firstly hopped on flat ground, and then some wood strips were placed on the ground to mimic the changing of terrain roughness. The estimated vertical foot forces and estimated contact time are shown in Figure 12. Three gait cycles on flat ground are shown in the figure, and the GMM predicted the contact time precisely.

As the GMM model was updated only when five new gait data had arrived, the GMM prediction performance degenerated when the terrain just changed. The leg force prediction module was evoked to detect the foot landing, leading to a detection time delay of about six control cycles. In the first updating phase of the GMM model, including the last gait cycle on flat ground and the first four gait cycles on rough terrain, there was only one gait cycle where the GMM predicted the foot contact. In the second GMM updating phase, the GMM successfully predicted the foot contact in two gait cycles. After two updating phases, the GMM could always predict the foot contact with a higher time delay of about two control cycles as the variance of the GMM gets higher, as shown in Table 3.

**TABLE 3:** Covariance matrices on flat ground and rough terrain.

Covariance matrix on flat ground	Covariance matrix on rough terrain
$\left[\begin{array}{ccc} 2.00 & -0.49 & 0.46\\ -0.49 & 1.83 & 1.04\\ 0.46 & 1.04 & 2.33 \end{array}\right]$	$\left[\begin{array}{ccc} 6.23 & -2.86 & -0.12\\ -2.86 & 3.07 & 0.05\\ -0.12 & 0.05 & 3.96 \end{array}\right]$

## Discussion

### Learning *Vs*. Data Fusion

Another effective contact detection approach is the data fusion technique, which combines different data to enhance accuracy. In the robot hopping experiments, the robot could detect the contact in one cycle delay, while the data fusion method applied to MIT cheetah 3 introduces a delay of four to five control cycles (Bledt et al., 2018a). This result shows that the proposed learning method can perform better if the robot runs on the same terrain with cyclic gait, which is true in most cases. Every animal or human would have preferred walking and running speeds, and the terrain types are limited in daily life. The gait contact parameters of hopping are distributed over a small area if the robot moves in the same terrain with the same gait.

### How Does the Learning Algorithm Adjust to Different Terrains to Achieve Fast Detection?

On flat ground, the elements in the covariance matrix would be small, and the prediction would be very accurate, while in rough terrain, the absolute values of the matrix elements increase, and the prediction becomes a little rough. The most important point is that the elements in the covariance matrix will go back to being small if the terrain is a flat ground again, which means that the learning prediction method can predict the contact as accurately as possible. Owing to the online learning method, the prediction algorithm can adapt to different terrains that the robot has never encountered before.

### Acquiring Data Online

A difficulty in learning the leg contact detection online is that the contact data for updating the GMM cannot be collected directly, as there is no force sensor to indicate the moment when the foot is just in contact with the ground. A “trace back” strategy is presented in this article. When the contact event is triggered by the GMM prediction module or leg force criterion, the update module will trace back the contact data in the memory stack until the data meet with the probability criterion.

### How Does Accurate Contact Prediction Affect Running Gait?

A state machine control strategy is usually adopted to control a legged robot’s running gait. A contact event will trigger a shift in state, and the control module will change as well. So an accurate detection of the contact event is critical, which is even more important in high-speed running. In our experiment, the control cycle is 4 ms, and the learning detection module can predict the contact event four control cycles in advance. In the fast running gait of a quadruped, the total gait cycle could be less than 300 ms, and the contact period for each leg can be less than 40 ms (Hudson et al., 2012). And the contact period includes the leg compressing phase and leg extending phase, with each period being 20 ms. So the proposed prediction method would ease the control strategy design and enhance the control performance.

## Conclusion

This paper presents a learning contact detection framework for legged robot running control. The algorithm continuously learns the characteristics of the contact data during robot running, including the period between two consecutive landing events, foot height, and contact force. A GMM is adopted to describe the three situations in running gait: running on flat ground, running upstairs, and running down stairs. Experimental results show that the mean value and covariance matrix of the contact data vector differ discernibly among different terrains, which enables learning detection.

To deal with the change in terrain and gait, an online learning scheme is presented. A main difficulty in online learning is acquiring contact data online without the help of force sensors. A “trace back” technique is proposed in this paper: when the contact event is triggered by the GMM or foot force threshold, the algorithm will trace back the contact data in the memory stack until the data meet with the probability criterion. And the GMM will be updated online after every five contact data points are collected, so the GMM can change with the terrain and gait. The learning contact detection algorithm was verified on a hopping robot. The detection model changed with the terrain by adapting the GMM parameters or, more specifically, by updating the mean value and covariance matrix of the sub-model in the GMM. Experimental results show that the learning algorithm can predict the foot contact to the ground four control cycles in advance compared with the detection method with only leg force. In the future, the algorithm will be applied to different robots and terrains to further verify its effectiveness, and more gait parameters can be added to the GMM to enhance prediction performance.

## Data Availability

The datasets presented in this study can be found in online repositories. The names of the repository/repositories and accession number(s) can be found below: https://github.com/Mr-Yuan0/Paper-code.
